# United States Veterinary Medical Association

**Published:** 1891-12

**Authors:** 


### EDITORIAL.

### UNITED STATES VETERINARY MEDICAL ASSOCIATION.

The following Committees have been appointed.

### Intelligence and Education.

Austin Peters..............23 Court St., Boston, Massachusetts.
E. C. Ross..............278 Elm St., New Haven, Connecticut.
P. Paquin..................................Battle Creek, Mich.
A. H. Baker..................145 Michigan Ave., Chicago, Ill.
Jas. A. Waugh.............59 E. Diamond St., Allegheny, Pa.

### Finance.

T. B. Rayner..................Chestnut Hill, Philadelphia, Pa.
Geo. H. Berns..............74 Adam St., Brooklyn, New York.
W. J. Coates.... ...... ....141 W. 54th St., New York City.

### Committee on Diseases.

A. W. Clement .... ..........W. Franklin St., Baltimore, Md.
J. F. Winchester ....................Lawrence, Massachusetts.
Jas. L. Kidd........... ......102 E. Main St., Lexington, Ky.
M. E. Knowles....... ................Terre Haute, Indiana.
Jno. Casewell.....................625 Madison St., Chicago, Ill

### Prize.

D. E. Salmon ... Washington, D. C.
D. J. Dixon ... New Jersey.
N. P. Hinckley ... Buffalo, New York.

### Army Legislation.

W. B. E. Miller ... 527 Penn St., Camden, New Jersey.
C. B. Michener ... 1303 Q St., N. W., Washington, D. C.
F. L. Kilborne ... Department of Agriculture, Washington.

### Publication.

W. H. Hoskins ... 12 S. 37th St., Philadelphia, Pa.
S. E. Weber ... Lancaster, Pensylvania.
W. S. Kooker ... 457 N. 4th St., Philadelphia, Pa.

### Special Committee on Food Inspection.

Olof Schwartzkopff, Cor. 4th & 14th Ave. S.E., Minneapolis, Minn.
G. C. Faville ... 210 Paul St., Baltimore.
W. Bryden ... 26 Sudbury St., Boston, Mass.

### Resident State Secretaries, 1891-92.

(No representative) ... California.
G. W. Pope ... 809 Government St., Mobile, Alabama.
Harrison Whitney ... Norwalk, Connecticut.
C. A. Cary ... Brookings, South Dakota.
H. P. Eves ... Wilmington, Delaware.
E. S. Walmer ... 3229 M St., Georgetown, D. C.
(No representative) ... Atlanta, Georgia
A. J. Thomson ... Terre Haute, Ind.
E. C. Hollingsworth ... La Salle, Illinois.
S. Stewart ... Council Bluffs, Iowa.
D. Lemay ... Fort Riley, Kansas.
James L. Kidd ... Lexington, Kentucky.
(No representative) ... Indian Territory.
J. L. Russell ... Orono, Maine.
L. H. Howard ... 1440 Washington St., Boston, Mass.
E. A. A. Grange ... Lansing, Michigan.
H. N. Waller ... Windom, Minnesota.
M. A. Piche ... Fort Custer, Montana.
John S. Meyer ... St. Joseph, Missouri.
A. W. Clement ... 111 W. Franklin St., Baltimore, Md.
T. P. Turner ... Fort Niobrara, Nebraska.
William H. Lowe ... Paterson, New Jersey.

W. T. Russell........................Nashua, New Hampshire.
Wm. R. Howe.. ...................... .........Dayton, Ohio.
Jno. Wende........................1593 Main St., Buffalo, N. Y.
C. D. McMurdo..................Fort Sill, Oklahoma Territory.
W. S. Kooker.... .............457 N. 4th St., Philadelphia, Pa.
Jas. A. Mclaughlin............1 Waterman St., Providence, R. I.
B, McInnes............. ..........Charleston, South Carolina.
J. W. Schiebler................312 3rd St., Memphis, Tennessee.
(No representative) ... ..............................Vermont.
John A. Myers.................... ......Harrisonburg, Virginia.
S. B. Nelson............. ........Spokane Falls, Washington.
E. D. Roberts..................... ......Janesville, Wisconsin.

FOREIGN CORRESPONDING SECRETARIES.

J. H. Frinck ............................................St. Johns, N. B

